# Managing large cutaneous squamous cell carcinoma with programmed cell death protein 1 inhibitor monotherapy: Real-world experience at a single center

**DOI:** 10.1016/j.jdin.2024.04.008

**Published:** 2024-05-03

**Authors:** Sach Thakker, Mairead R. Baker, Jafar Al-Mondhiry, Sekwon Jang, Jennifer A. DeSimone

**Affiliations:** a Georgetown University School of Medicine, Washington, District of Columbia; b Department of Dermatology, MedStar Washington Hospital Center, Washington, District of Columbia; c Inova Dwight and Martha Schar Cancer Institute, Fairfax, Virginia

**Keywords:** cemiplimab, cutaneous oncology, immunotherapy, PD-1 inhibitor, pembrolizumab, squamous cell carcinoma

*To the Editor:* Cutaneous squamous cell carcinoma (cSCC) is an immunogenic malignancy with high mutational burden and significantly increased incidence in immunocompromised populations, rendering it an exceptional target for immune checkpoint inhibitor (ICI) therapy.^1^ Programmed cell death protein 1 (PD-1) inhibitors are approved for the treatment of advanced or metastatic cSCC in patients who are poor candidates for curative surgery or radiation.^2^^,^^3^ Tumor diameter >2cm uniformly confers upstaging and poorer prognosis and is the risk factor most highly associated with disease-specific death.^4^

For this report, we performed a retrospective review of 54 patients with cSCC tumors over 3 cm in size treated with either pembrolizumab or cemiplimab at InovaSchar Cancer Institute between January 2019 and April 2023. Inclusion criteria consisted of adult age and over 3 cm locally advanced or metastatic cSCC tumors, such as seen in Fig 1. Selected patients either had inoperable tumors due to size including depth or were deemed poor surgical/curative radiation candidates based on overall health and/or anticipated loss of function/morbidity posttreatment. Demographic data, clinical findings, adverse events, and patient response to PD-1 inhibitors were recorded. Primary end points were overall response rate, complete response, partial response, stable disease, and progression of disease which were evaluated using the RECISTv1.1 criteria. The primary analysis was estimated with its 2-sided 95% confidence interval using the Clopper-Pearson method.

**Fig 1:**
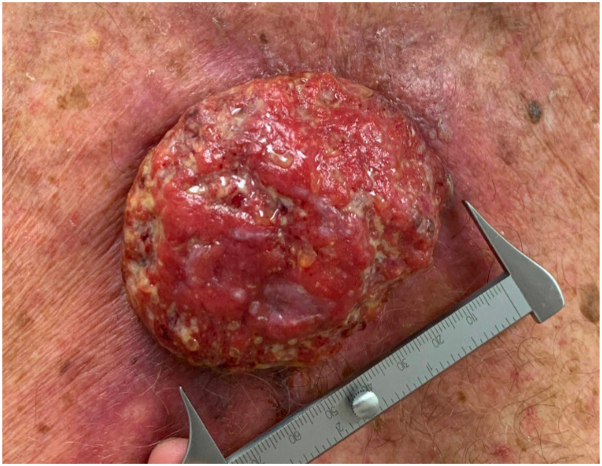
Clinical example of a well-differentiated locally advanced 7.6 cm cutaneous squamous cell carcinoma of the right upper chest before treatment with pembrolizumab.

In patients treated with pembrolizumab (400 mg intravenous every 6 weeks), 54% (19) had complete response, 20% partial response (7), 9% progression of disease (3), 3% stable disease (1), and 14% (5) were not evaluable. The mean and median number of infusions was 4.8 and 4, respectively. Seven patients (20%) reported an immune-related adverse event, with one discontinuing treatment due to myocarditis. The overall response rate of the pembrolizumab treatment group was 74% (95% confidence interval 57%-88%), which was significantly higher than the historical control, 41% (*P* = .004).^2^ In patients treated with cemiplimab (350 mg intravenous every 3 weeks), 58% (11) had complete response, 5% partial response (1), 16% progression of disease (3), 5% stable disease (1), and 16% (3) were not evaluable. The mean and median number of infusions was 7.4 and 6, respectively. Six patients (32%) reported an immune-related adverse event, with one discontinuing treatment due to elevated liver function fests. The overall response rate of the cemiplimab treatment group was 63% (95% confidence interval 38%-84%), which was similar to the historical control 47% (*P* = .183) (Table I).^3^

**Table I:** Patient characteristics and treatment response

Variable	Total cohort (*n* = 54)	Pembrolizumab (*n* = 35)	Cemiplimab (*n* = 19)
Sex			
Males	33 (61%)	22 (63%)	11 (58%)
Females	21 (39%)	13 (37%)	8 (42%)
Mean diameter (cm)	5.1 (3-8)	5.0 (3-8)	5.3 (3-8)
Mean age and range (years)	77.9 (50-98)	77.3 (50-98)	78.9 (55-96)
Mean number of infusions	5.7 (1-17)	4.8 (1-17)	7.4 (1-15)
Median number of infusions	5	4	6
Stage			
Locally advanced	36 (67%)	21 (60%)	15 (79%)
Metastatic	18 (33%)	14 (40%)	4 (21%)
Type of tumor			
Primary	29 (54%)	17 (49%)	12 (63%)
Recurrent	25 (46%)	18 (51%)	7 (37%)
Average time to follow-up (d)	535	462	654
Immune-related adverse events (irAEs)	13 (24%)	7 (20%)	6 (32%)
Discontinued treatment	2 (4%)	1 (3%)	1 (5%)
Primary tumor location			
Head and neck	39 (72%)	25 (72%)	14 (74%)
Trunk	6 (11%)	4 (11%)	2 (10%)
Extremities	9 (17%)	6 (17%)	3 (16%)
Histologic differentiation			
Well	20 (37%)	15 (43%)	5 (26%)
Moderate	22 (41%)	13 (37%)	9 (48%)
Poor	12 (22%)	7 (20%)	5 (26%)
Outcome			
Complete response (CR)	30 (55%)	19 (54%)	11 (58%)
Partial response (PR)	8 (15%)	7 (20%)	1 (5%)
Progression (PD)	6 (11%)	3 (9%)	3 (16%)
Stable disease (SD)	2 (4%)	1 (3%)	1 (5%)
Not evaluable (NE)	8 (15%)	5 (14%)	3 (16%)
Overall response rate (ORR)	70%	74%	63%

It has been widely reported that ICIs are successful in treating inoperable cSCC.^2^^,^^3^ In this report, we focused on the challenge of managing large tumors and demonstrated that PD-1 inhibitor monotherapy achieved complete disease control in better than 50% of patients, with rapid tumor destruction and few total infusions. Long-term durability of response remains to be assessed as our study only follows patients 1 to 2 years posttreatment. Standardization of treatment end point and determination of ideal duration of treatment are warranted.

Recent observations have suggested that while ICIs are efficacious in promoting local destruction of primary cSCCs, they perplexingly do not reduce the total burden of epidermal atypia as patients can retain or develop new actinic keratoses or cSCC while on immunotherapy.^5^ This underscores the need for frequent monitoring of these patients, independent of magnitude of ICI response.

## Conflicts of interest

None disclosed.
